# Evaluation of kidney function and damage in patients with hemophilia B—insights from the B-Natural study

**DOI:** 10.1016/j.rpth.2025.103181

**Published:** 2025-09-17

**Authors:** Jan Astermark, Petra LeBeau, Anders Christensson, Stefan Lethagen, Erik Berntorp, Amy D. Shapiro, B-Natural Study investigators

**Affiliations:** 1 Department of Translational Medicine, Lund University, Malmö, Sweden; 2 Department of Hematology, Oncology and Radiation Physics, Skåne University Hospital, Malmö, Sweden; 3 Rho, Inc, Durham, North Carolina, USA; 4 Department of Clinical Sciences, Lund University, Malmö, Sweden; 5 Sobi, Stockholm, Sweden; 6 Clinical Coagulation Research, Department of Translational Medicine, Lund University, Malmö, Sweden; 7 Indiana Hemophilia and Thrombosis Center, Indianapolis, Indiana, USA

**Keywords:** hemophilia B, factor IX deficiency, hypertension, kidney disease, renal insufficiency

## Abstract

**Background:**

Patients with hemophilia are reported to have a high prevalence of established and potential risk factors for kidney dysfunction and damage. However, comprehensive studies specifically evaluating kidney function in hemophilia, particularly hemophilia B (HB), are limited, with most research focusing on hemophilia A.

**Objectives:**

This study aimed to assess markers of glomerular filtration, damage, and tubular function in patients with HB enrolled in the B-Natural study.

**Methods:**

Kidney function and damage was evaluated using estimated glomerular filtration rate (eGFR), urine albumin/creatinine ratio, urinary immunoglobulin/creatinine ratio, and protein HC. Correlations with patient characteristics, treatment regimes, and comorbidities were analyzed.

**Results:**

The cohort consisted of 209 patients with HB, with 32% having severe, 51% moderate, and 17% mild disease. The median age was 13 years (IQR, 9-22 years; range, 1-73 years). The mean eGFR across the cohort was 107 mL/min/1.73 m^2^ (range, 13-183 mL/min/1.73 m^2^). Older age, higher body mass index, a history of kidney disease, diabetes, hypertension, and Asian ethnicity were significantly associated with lower eGFR. Patients with severe HB (factor IX < 1%), and/or a history of inhibitors exhibited a wider range of eGFR values. Only a small proportion of patients showed glomerular damage and tubular dysfunction. Regular prophylactic treatment with factor concentrates had no apparent impact on kidney function or kidney disease markers.

**Conclusion:**

Our findings suggest that patients with HB and no inhibitors do not have an increased risk of kidney dysfunction or damage compared to persons without hemophilia. Age and hypertension were the primary risk factors, underscoring the importance of regular follow-ups.

## Essentials

• Data on kidney function and the risk of kidney disease in hemophilia B (HB) are limited.
• Markers of glomerular filtration, damage, and tubular function were assessed in persons with HB.
• Regular replacement therapy appeared to have no impact on kidney function.
• No increased risk of kidney disease was found in White patients without inhibitors.

## 1 Introduction

Hemophilia B (HB) is a congenital X-linked bleeding disorder affecting mainly males. The disease is characterized by a deficiency of coagulation factor (F)IX requiring life-long treatment to prevent and to treat deleterious bleeds [1]. Typically, bleeds occur in the joints but may appear in any tissue with the potential of causing complications and comorbidities including involving the kidneys. In a review from the 1990s of the medical records of 3422 patients with hemophilia A (HA) or HB in the United States, 2.9% were diagnosed with either acute renal disease and/or chronic renal disease (CRD). Patients with hemophilia (PWH) who had CRD were more likely to be older, non-White and to have had a recent admission for a kidney bleed than those without diagnosed CRD. In addition, in a multivariate analysis, HIV infection and hypertension were strongly associated with both acute renal disease and CRD [2].

Hematuria is relatively frequently experienced in hemophilia, but its long-term effect on the kidney and kidney function is subject to debate [3]. The same is true for urolithiasis, which seems to be significantly more prevalent in PWH compared with the general population (odds ratio [OR], 23.4; under 40 years of age) [4]. Interestingly, children with hemophilia also seem to have higher urinary calcium excretion, more intermittent hypercalciuria, and lower whole body bone mineral density than controls despite prophylaxis, and this may potentially contribute to hypertension, nephrocalcinosis, and impact upon kidney function in adulthood. However, hypercalciuria was not associated with age, severity of hemophilia, or previous hypercalciuria [5].

Hypertension is a strong established risk factor for kidney disease, and although divergent results have been published within the hemophilia population, the reported prevalence of hypertension is usually considered to be higher than the general population [6]. The reason(s) for this, however, have not been settled, and even the usual cardiovascular risk factors have not made it possible to account for the higher prevalence of hypertension in PWH [6]. In a multivariate analysis of 135 adult males with mild to severe HA and HB, only age was a significant predictor of kidney disease, whereas hematuria, hypertension, kidney insufficiency or hemophilia severity were not [7]. Finally, hypertension was diagnosed in 239 patients (45%) at centers taking part in the Advance network and was associated with age and body mass index (BMI; adjusted OR, 18.1 and 25.1), eGFR of <70 mL/min/1.73 m^2^ (OR, 2.7), diabetes (OR, 2.8), coronary artery disease (OR, 3.3), and a family history of hypertension (OR, 4.4) but was not associated with severity of hemophilia or history of hematuria [3].

Much remains to be explored regarding kidney function and the risk of kidney disease in PWH, in both patients with HA and those with HB. In addition, significant differences exist between the 2 types of hemophilia and all studies, in this disease area as well as in others, mainly include patients with HA, with and without regular prophylactic treatment, and various bleeding phenotypes. Studies focusing on HB are warranted. Hence, the B-Natural (BN) study cohort was established to shed more light on the HB disease, including kidney function. The BN study is a multicenter, international, observational study of HB including both retrospectively and prospectively collected data, designed to increase understanding of clinical manifestations, treatment, quality of life, inhibitor development, immune tolerance induction (ITI) outcome, kidney function, and create a biorepository for future investigations [8]. The objective of the current study is to describe the observed kidney function in terms of filtration capacity over time indicated by the estimated glomerular filtration rate (eGFR), minor and major kidney damage identified by urine albumin:creatinine ratio (UACR) and urine immunoglobulin/creatinine ratio (UIGCR), respectively, as well as tubular dysfunction assessed by protein HC (also known as ɑ1-microglobulin) in patients HB. Correlations with demographic and clinical characteristics, treatment received, inhibitor status, ITI therapy performed, and comorbidities will also be explored.

## 2 Methods

### 2.1 BN study overview

Demographics of the BN cohort have been described previously [8]. Subjects were eligible to participate if they had FIX deficiency, any level of severity, and were part of an affected sibling pair/group; and/or had a current or history of inhibitor, defined as >0.6 Bethesda units determined by the local laboratory. In total, 224 individuals from 107 families were enrolled from 24 centers in North America (*n* = 16), Europe (*n* = 7) and Asia (*n* = 1). Sixty-eight (30.4%) subjects had severe (<1 IU/dL), 114 (50.9%) moderate (1-5 IU/dL), and 42 (18.8%) mild (>5 to <40 IU/dL) disease. Twenty-nine participants had inhibitors or a history of inhibitors, all of whom had severe disease. The study included 4 female subjects, all with mild disease. Age distributions as measured by the median (25th, 75th percentile) for the severe, moderate, and mild disease severity groups were 15.4 (11.0, 32.3), 13.3 (8.58, 20.3), and 12.1 (7.65, 20.8) years, respectively, and 16.5 (8.08; 31.9) years for the group with inhibitors. Few individuals above 50 years of age were enrolled whereas the age distribution below 50 was rather even across the groups. Participants were followed for at least 6 months with physical examination, for example, blood pressure, performed at the follow-up visit and treatment-related factors including bleed rate, inhibitors, and comorbidity recorded. The medical and family history included information related to allergies, use of medications, and chronic diseases. If a history of kidney disease was indicated, additional details were collected, such as the diagnosis (ie, renal failure, glomerulonephritis, tubulointerstitial nephritis, nephrosis, or any other specified kidney disease), and current or past treatment. If the participant had a history of an inhibitor, additional information including characteristics of the inhibitor, treatment provided, and outcome were reported. The incidence of subclinical or clinical expression of kidney disease was determined at study entry by measurement of the UACR and UIGCR and by urinary protein electrophoresis. Steady-state kidney function was determined by eGFR based on plasma cystatin C. The procedures followed were approved by the ethical committees in each participating center. BN is registered at ClinicalTrials.gov (NCT02502409).

### 2.2 Specimen collection and processing

At study entry, a spot urine sample (10 mL) and a blood specimen were collected. If a urine sample was cloudy, it was first centrifuged. Urine samples were then frozen and stored at the center until all subjects at the site had completed the study and were then shipped to the central laboratory. The urine specimens were evaluated for albumin, creatinine, immunoglobulin G, and protein HC. The plasma level of cystatin C was determined by an automated particle-based immunoassay, adjusted to the international reference preparation ERM-DA 471/IFCC [9]. The plasma levels of creatinine were measured with enzymatic colorimetric assays and calibrated to IDMS [10]. All urine and plasma analyses were centrally performed using routine accredited methods at Skåne University Hospital Malmö/Lund.

### 2.3 Data derivations

Relative eGFR was calculated using the cystatin-C–based CAPA equation [9], which is appropriate for both children and adults and different ethnicities, as follows:

$$eGFR=130\;x\;CystatinC^{-1.069}\;x\;age^{-0.117}-7$$

eGFRs calculated by the abovementioned equation were further categorized by use of the 2024 KDIGO classification [11], in milliliters per minute per 1.73 m^2^—G1, ≥90 (normal or high); G2, 60 to 89 (mildly decreased); G3a, 45 to 59 (mildly to moderately decreased); G3b, 30 to 44 (moderately to severely decreased); G4, 15 to 29 (severely decreased); and G5, <15 (kidney failure).

Albuminuria categories were created from the UACR by use of the 2024 KDIGO classification [11], in milligrams per millimoles—A1, <3 (normal to mildly increased); A2, 3 to 30 (moderately increased); and A3, >30 (severely increased).

Guidelines from the American Heart Association (AHA) were used to categorize blood pressure values measured in millimeters per mercury—systolic [S] and diastolic [D] at the enrollment visit [12]—normal: <120 (S) and <80 (D) mm Hg; elevated: 120 to 129 (S) and <80 (D) mm Hg; hypertension-I (HTN-I), 130 to 139 (S) or 80 to 89 (D) mm Hg; and hepertension-II (HTN-II), 140 mm Hg or higher (S) or 90 mm Hg or higher (D).

### 2.4 Statistical analysis

The analysis population for this study consisted of all enrolled male participants with nonmissing relative eGFR. All statistical analyses were performed using R Statistical Software (v4.3.1; R Core Team) [13], and a *P* value of <.05 was considered significant. To compare differences in demographics and clinical characteristics between disease severity groups, Chi-square and Fisher exact tests were used for categorical variables and the Kruskal-Wallis test by ranks for continuous variables. Normality of renal end points were explored using histograms with density curves and quantile-quantile (qq) plots.

Since the barrier damage (UACR and UIGCR) and tubular dysfunction (protein HC) markers had distributions that were highly right-skewed, we summarized results using median and IQR and used ranked regression to analyze the relationship between the damage markers and the patient demographics or clinical characteristics. In this context, the β coefficients obtained from ranked regression are equivalent to the Spearman rank-order correlation coefficients, providing a measure of the monotonic relationship between ranked variables.

Relationships between normally distributed kidney function (eGFR) and participant demographics or clinical characteristics were examined by means of scatterplots, the Pearson correlation coefficient as a measure of strength of association, and univariate and multivariate linear regression. Univariate models allowed us to examine the effect of each risk factor independently, while the multivariate model assessed the combined effect of all risk factors simultaneously.

For regression models, missing blood pressure categories from 10 subjects were imputed with the value normal, which is a conservative approach based on the facts that (1) these participants reported no history of hypertension; (2) the majority of participants without a history of hypertension had blood pressure categories in the normal range at the time of visit (80.4%); and (3) these participants were 1 to 7 years of age and therefore less likely to have hypertension, although it is possible. Without imputation, the records of participants with missing blood pressure category would otherwise be removed in its entirety when building regression models. This would lead to the loss of a substantial amount of data and therefore the precision of the estimators would be lower; additionally, this could lead to a biased representation of the original data (eg, in case the missing process is associated with the values of the response or predictors). Furthermore, model selection could be problematic with missing data, since the number of complete cases changes with the addition and removal of predictors. As the type of hypertension medication was strongly correlated with a history of hypertension (and similarly, a history of kidney disease and its treatment), the associations of these individual treatments with kidney function were not explored in any of the regression models.

## 3 Results

### 3.1 Patient characteristics

The original 224 enrolled participants from the BN cohort were subset to meet the analysis population criteria for this study (ie, male and nonmissing eGFR). Four female subjects and 11 subjects with missing eGFR (2 with severe, 8 with moderate, and 1 with mild FIX deficiency) were excluded, resulting in 209 participants. Table 1 shows the demographic and clinical characteristics of this population by their FIX deficiency disease severity. Sixty-six (32%) had severe FIX deficiency, 106 (51%) moderate, and 37 (17%) mild. Twenty-eight had a current or history of an inhibitor, and all of these had severe disease. The median age was 13 years (IQR, 9-22 years, range, 1-73 years) with about two-thirds of the participants younger than 18 years of age (64%). Most participants were from North America (73%) and of White ethnicity (80%).

**Table 1:** Patient demographics and clinical characteristics by disease severity group.

Characteristic	Overall (*N* = 209)^a^	Severity group				*P*
		Inhibitor (*n* = 28)^a^	No inhibitor: severe (<1%) (*n* = 38)^a^	No inhibitor: moderate (1%-5%) (*n* = 106)^a^	No inhibitor: mild (>5%) (*n* = 37)^a^	
Age group						.15^b^
Child	134/209 (64)	14/28 (50)	22/38 (58)	70/106 (66)	28/37 (76)	
Adult	75/209 (36)	14/28 (50)	16/38 (42)	36/106 (34)	9/37 (24)	
Age (y)						.18^c^
No. missing	0	0	0	0	0	
Mean (SD)	18 (15)	24 (20)	22 (17)	17 (12)	16 (14)	
Median (IQR)	13 (9-22)	19 (10-33)	15 (10-29)	13 (8-20)	11 (7-17)	
Range	1-73	1-73	2-67	1-68	3-62	
Continent						**<.001**^b^
North America	152/209 (73)	16/28 (57)	21/38 (55)	86/106 (81)	29/37 (78)	
Europe	38/209 (18)	12/28 (43)	10/38 (26)	8/106 (7.5)	8/37 (22)	
Asia	19/209 (9.1)	0/28 (0)	7/38 (18)	12/106 (11)	0/37 (0)	
Ethnicity						**<.001**^b^
Asian	20/209 (9.6)	0/28 (0)	7/38 (18)	13/106 (12)	0/37 (0)	
Black	15/209 (7.2)	5/28 (18)	8/38 (21)	2/106 (1.9)	0/37 (0)	
White	168/209 (80)	21/28 (75)	23/38 (61)	89/106 (84)	35/37 (95)	
Other	6/209 (2.9)	2/28 (7.1)	0/38 (0)	2/106 (1.9)	2/37 (5.4)	
BMI (kg/m^2^)						.37^c^
No. missing	0	0	0	0	0	
Mean (SD)	21 (7)	24 (8)	22 (8)	21 (6)	21 (6)	
Median (IQR)	20 (16-24)	22 (18-26)	20 (17-27)	20 (16-24)	20 (16-25)	
Range	13-44	15-42	14-42	13-44	14-35	
History of renal disease						**.009**^b^
No	193/209 (92)	22/28 (79)	34/38 (89)	100/106 (94)	37/37 (100)	
Yes	16/209 (7.7)	6/28 (21)	4/38 (11)	6/106 (5.7)	0/37 (0)	
History of diabetes						**.028**^b^
No	207/209 (99)	28/28 (100)	36/38 (95)	106/106 (100)	37/37 (100)	
Yes	2/209 (1.0)	0/28 (0)	2/38 (5.3)	0/106 (0)	0/37 (0)	
History of hypertension						**<.001**^b^
No	188/209 (90)	20/28 (71)	31/38 (82)	100/106 (94)	37/37 (100)	
Yes	21/209 (10)	8/28 (29)	7/38 (18)	6/106 (5.7)	0/37 (0)	
Blood pressure category (per AHA)						.092^b^
Normal	112/199 (56)	12/28 (43)	20/38 (53)	59/99 (60)	21/34 (62)	
Elevated	35/199 (18)	5/28 (18)	4/38 (11)	22/99 (22)	4/34 (12)	
HTN-I	37/199 (19)	7/28 (25)	9/38 (24)	12/99 (12)	9/34 (26)	
HTN-II	15/199 (7.5)	4/28 (14)	5/38 (13)	6/99 (6.1)	0/34 (0)	
History of ITI						>.99^d^
No	8/28 (29)	8/28 (29)	0/0 (NA)	0/0 (NA)	0/0 (NA)	
Yes	20/28 (71)	20/28 (71)	0/0 (NA)	0/0 (NA)	0/0 (NA)	
History of smoking (adults only)						.49^b^
Never	47/75 (63)	5/14 (36)	11/16 (69)	25/36 (69)	6/9 (67)	
Past	10/75 (13)	3/14 (21)	2/16 (13)	4/36 (11)	1/9 (11)	
Current	18/75 (24)	6/14 (43)	3/16 (19)	7/36 (19)	2/9 (22)	
Treatment in year prior to enrollment						**<.001**^b^
Prophylaxis	59/209 (28)	24/28 (86)	25/38 (66)	8/106 (7.5)	2/37 (5.4)	
On-demand	150/209 (72)	4/28 (14)	13/38 (34)	98/106 (92)	35/37 (95)	
ACE inhibitor						**.036**^d^
No	203/209 (97)	26/28 (93)	35/38 (92)	105/106 (99)	37/37 (100)	
Yes	6/209 (2.9)	2/28 (7.1)	3/38 (7.9)	1/106 (0.9)	0/37 (0)	
Angiotensin receptor blocker						0.21^b^
No	207/209 (99)	27/28 (96)	37/38 (97)	106/106 (100)	37/37 (100)	
Yes	2/209 (1.0)	1/28 (3.6)	1/38 (2.6)	0/106 (0)	0/37 (0)	
NSAID						**<.001**^b^
No	196/209 (94)	21/28 (75)	36/38 (95)	103/106 (97)	36/37 (97)	
Yes	13/209 (6.2)	7/28 (25)	2/38 (5.3)	3/106 (2.8)	1/37 (2.7)	
Antiretroviral						.21^b^
No	207/209 (99)	27/28 (96)	37/38 (97)	106/106 (100)	37/37 (100)	
Yes	2/209 (1.0)	1/28 (3.6)	1/38 (2.6)	0/106 (0)	0/37 (0)	

Bold values denote statistical significance at the *P* < .05 level.

ACE, angiotensin-converting enzyme; AHA, American Heart Association; BMI, body mass index; HTN, hypertension; ITI, immune tolerance induction; NSAID, nonSteroidal anti-inflammatory drug.

a Continuous variables: mean (SD), median (IQR), and range; categorical variables: *n*/*N* (%).

b Pearson Chi-squared test.

c Kruskal-Wallis rank-sum test.

d Fisher exact test.

Two participants reported a history of diabetes, both with severe disease. Twenty-one (10%) reported a history of hypertension, and 16 of these were under treatment for hypertension at the time of evaluation. Sixteen (8%) reported a history of kidney disease: nephrolithiasis (*n* = 7), nephrotic syndrome (*n* = 5), renal cyst (*n* = 1), renal insufficiency (*n* = 2), and tubulointerstitial nephritis (*n* = 1). Most (*n* = 6) participants with nephrolithiasis had moderate FIX disease, whereas all others with kidney disease had severe FIX disease. Most participants never smoked (87%), which is partly a reflection of the large pediatric population in this study. When subset for adults only, no difference was observed in the history of smoking categories between disease severity groups (*P* = .5). Treatment in the year prior to enrollment was mostly on-demand (72%) and as expected primarily in participants with moderate and mild disease, whereas prophylaxis was mainly used in severe disease. For those with an inhibitor, 20 (71%) had a history of ITI, and these patients have been reported previously [14]. Most study participants had either normal or elevated blood pressure measurements at the study visit (*n* = 112, 56% and *n* = 35, 18%, respectively), whereas about a quarter of the participants had readings indicative of hypertension: HTN-I (*n* = 37, 19%) or HTN-II (*n* = 15, 7.5%). The analysis population for the minor and major kidney damage and tubular dysfunction end points was 185 participants, due to missing urinalysis data for 24 of the 209 participants.

### 3.2 Glomerular filtration rate

**Figure 1:**
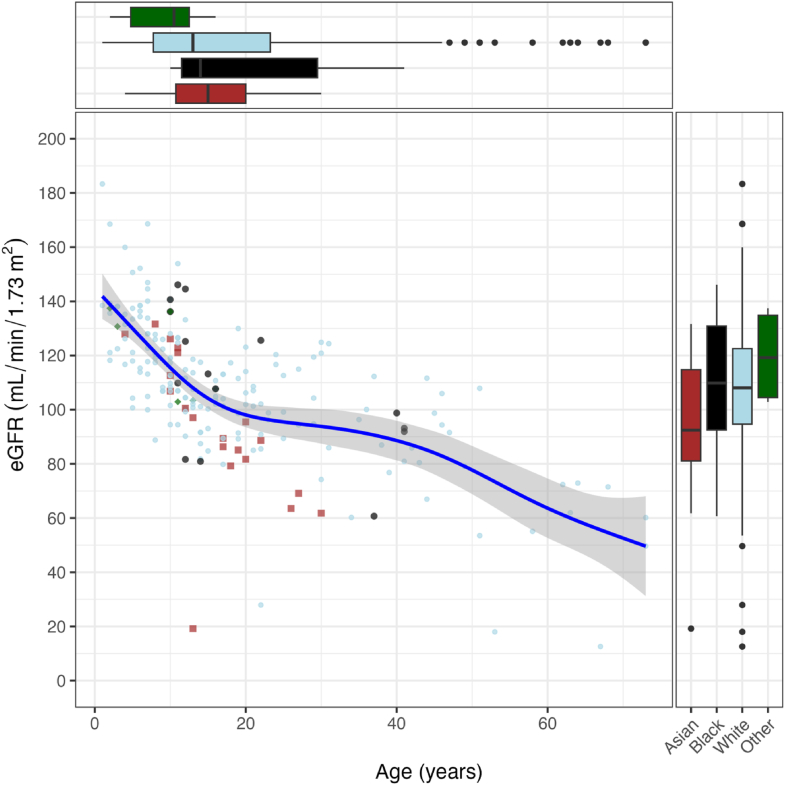
Relationship between eGFR, age, and ethnicity. The figure illustrates the relationship between age and eGFR among different ethnic groups. Each point represents an individual, with colors and shapes distinguishing ethnicities. The blue smoothed line indicates the general trend across all data points. Boxplots for ethnicity are shown along the axes to represent the distribution of this variable in relation to age and eGFR. eGFR, estimated glomerular filtration rate.

The mean eGFR (SD) in the study population was 107 (26) mL/min/1.73 m^2^, with a range of 13 to 183 mL/min/1.73 m^2^ and did not show any obvious deviations from the general population [15,16]. The smoothed line describing the relationship between eGFR and age is shown in Figure 1. The Pearson correlation coefficient was significant (*R* = −0.66; *P* < .01), indicating a strong linear decrease in eGFR with increasing participants’ ages. However, the nonlinear curve showed a steep decline for children from the age of 1 until 18 years, followed by a plateau between 18 and about 40 years of age, after which the curve started to slowly decline again. Importantly, most of the participants in our cohort were <18 years old, resulting in fewer data points for adults, which is also indicated by a wider 95% CI for the adults. Seven participants (1 child and 6 adults) had eGFR measurements less than 60 mL/min/1.73 m^2^, indicating a potential kidney disorder. All 7 subjects had blood pressure measurements in the HTN-I and HTN-II categories at the time of study entry. The child was a 13-year-old Asian with an eGFR of 19 mL/min/1.73 m^2^ and severe HB disease without FIX inhibitor nor any reported kidney disease, and who received on-demand FIX treatment. The 6 adults were Whites with ages ranging from 22 to 73 years old and had severe disease. Three of these patients had a history of FIX inhibitors and ITI procedures. Three patients had kidney disease, for example, renal insufficiency or tubulointerstitial nephritis, one of whom had diabetes and was a current smoker. All were taking medication for hypertension, and all but 1 was on prophylaxis. Asians had the lowest eGFR measurements compared with any of the other ethnicities in our study, despite most Asians were of a younger age. Importantly, however, the number of subjects in the Asian and Black ethnicity groups was relatively small in our study (9.6% and 7.2%, respectively), and the methods used for eGFR have not been validated for all ethnicities. Most of the Asian subpopulation had normal blood pressure (70%); did not have a history of hypertension (95%), kidney disease (100%), or diabetes (100%); and never smoked (90%). All Asians had severe or moderate hemophilia and all but one received on-demand FIX treatment.

**Figure 2:**
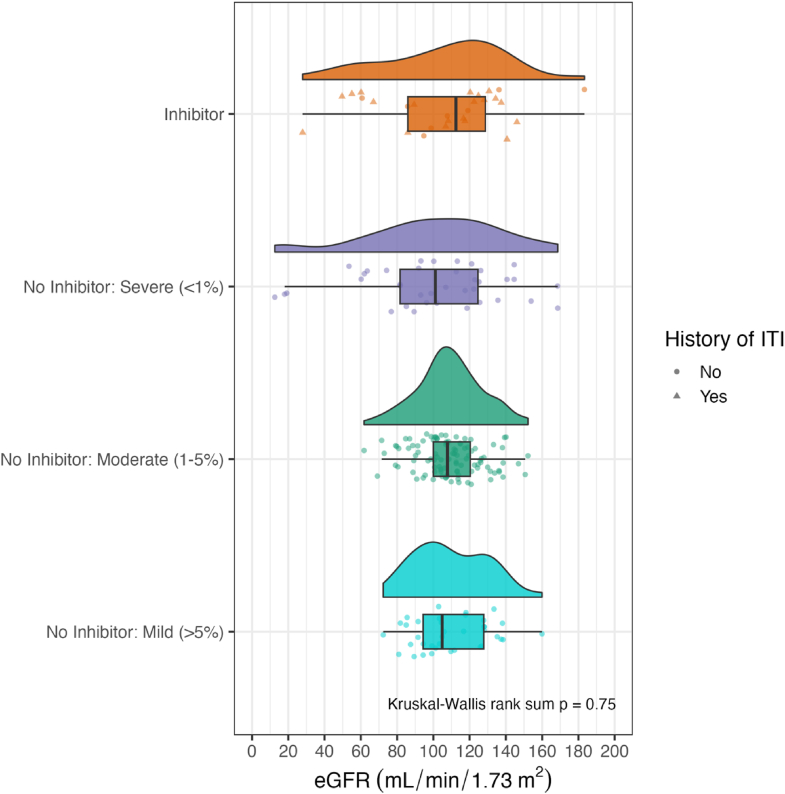
Distribution of eGFR by disease severity group and a history of ITI. The figure shows distribution curves (density plots) of eGFR for each disease severity group. Each density plot is accompanied by a boxplot below it to represent the distribution and variability of eGFR within each severity group. Individual data points are marked with different symbols to indicate the presence or absence of a history of ITI. eGFR, estimated glomerular filtration rate; ITI, immune tolerance induction.

The distribution of the eGFR values in patients of different severity groups and in those with a history of inhibitors are shown in Figure 2. The inhibitor and severe disease groups had a wider range of eGFR values than the moderate and mild severity groups, although no significant differences were observed between median eGFR of the 4 severity subgroups (*P* = .75).

**Figure 3:**
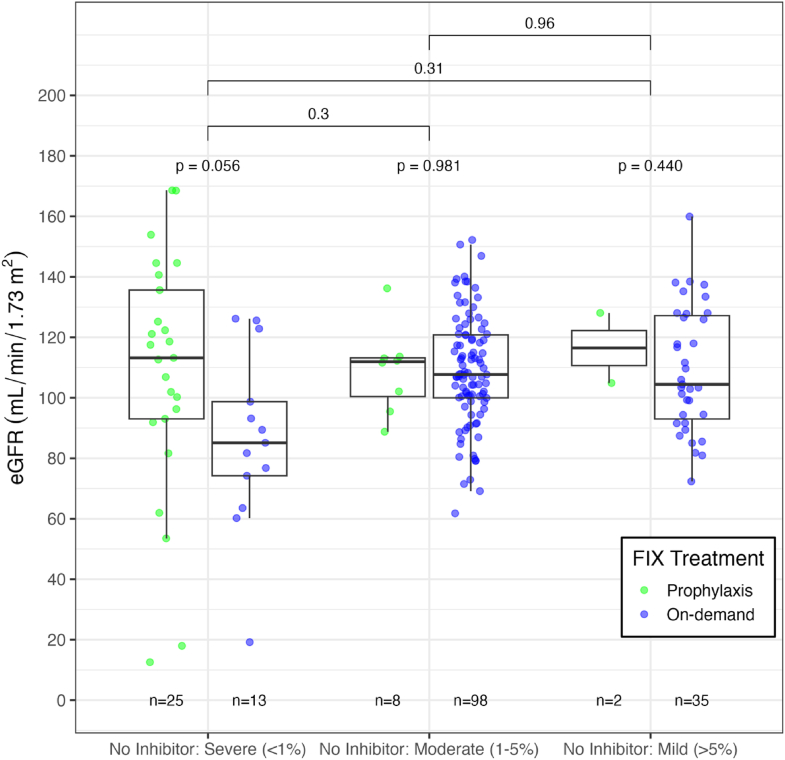
Boxplot of eGFR by disease severity group and treatment type (prophylaxis or on-demand) for patients without inhibitors. This figure shows boxplots of eGFR for each disease severity group further broken down by treatment type (prophylaxis or on-demand), resulting in 2 boxplots per severity group. The analysis includes patients with no inhibitors only. Prophylaxis and on-demand treatment are compared both between and within each severity group. Associated *P* values are indicated on the figure and were calculated using the Wilcoxon rank-sum test.

Figure 3 shows the distribution of eGFR measurements within each disease severity group and by treatment received in the year prior to enrollment (ie, prophylaxis vs on-demand) and only includes participants without a history of FIX inhibitor. Overall comparison between those who received prophylaxis in the year prior to enrollment with those who received on-demand treatment was not significant (*P* = .32, not shown). No significant differences were observed within and across each of the severity groups, although the on-demand treated subjects within the severe disease group tended to have lower eGFR values (*P* = .056). Within participants with FIX inhibitors, 3 of 20 subjects with a history of ITI had eGFR values of <60 mL/min/1.73 m^2^. These 3 subjects had histories of hypertension. In addition, 1 subject had a diagnosis of tubulointerstitial nephritis, received on-demand FIX treatment, and was taking a calcium channel blocker. The other 2 subjects were on prophylaxis and did not have a known kidney disease, but 1 was a smoker, the other a past smoker; and 1 was taking a β-blocker and the other an angiotensin receptor blocker. Importantly, all 5 cases of nephrotic syndrome in the study cohort were reported in patients with inhibitors having performed ITI procedures.

**Figure 4:**
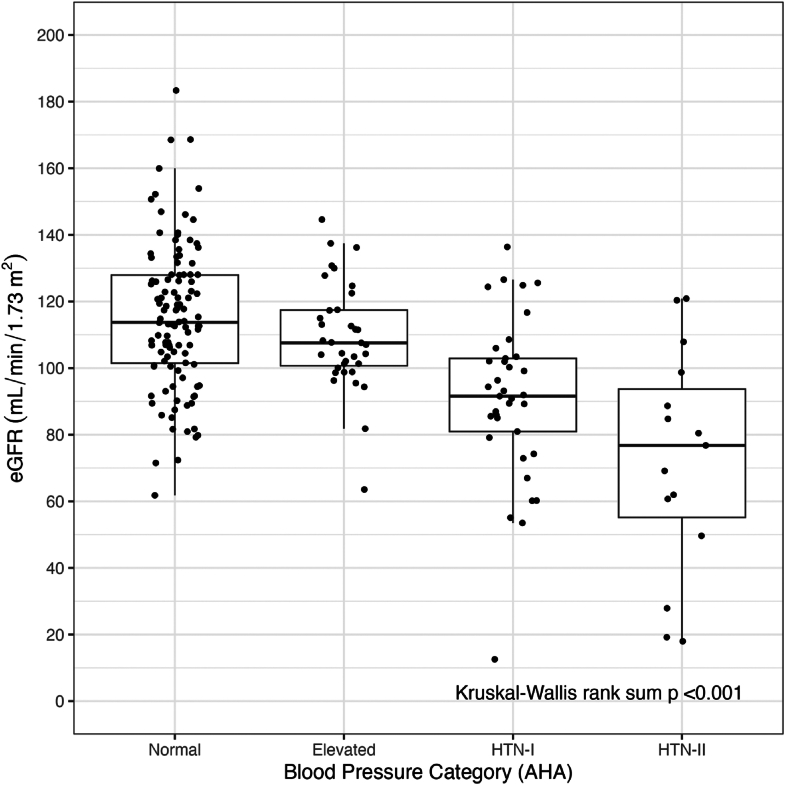
Boxplot of eGFR by blood pressure category (per AHA). The figure displays boxplots of eGFR for each blood pressure category as defined by the AHA. The overall *P* value for the comparison across all blood pressure categories is displayed. AHA, American Heart Association; eGFR, estimated Glomerular Filtration Rate; HTN-I, Hypertension-I; HTN-II, Hypertension-II.

The relationship between eGFR and the AHA blood pressure category at study visit is shown in Figure 4. The eGFR values were significantly different between the blood pressure groups (Kruskal-Wallis *P* < .001), and subjects with hypertension had lower eGFR values than those with normal or elevated blood pressure. Table 2 summarizes the results from univariate linear regression models and the multivariate model, exploring the associations of predetermined clinical risk factors with eGFR and included age, nonlinear age, BMI, ethnicity, a history of kidney disease, a history of diabetes, a history of hypertension, blood pressure classification, disease severity group, and the use of NSAIDs. Apart for disease severity and the use of NSAIDs, all other risk factors were significant predictors of eGFR in the univariate models. When combining all risk factors in a multivariate regression model, the following clinical variables were significantly associated with lower values of eGFR: older age (nonlinear relationship), Asian ethnicity (compared with the other ethnicities), a history of diabetes, and a history of hypertension. Use of NSAIDs, while controlling for all other risk factors in the model, showed a possible relationship with higher eGFR measurements (*P* = .076). BMI, a history of kidney disease, blood pressure classification at enrollment, and disease severity group were not significant when controlling for the other risk factors.

**Table 2:** Linear univariate and multivariate regression models for eGFR and pre-selected clinical risk factors.

Characteristic	Descriptive	Univariate models				Multivariate model			
	*N* = 209^a^	Forest plot	β	95% CI	*P*	Forest plot	β	95% CI	*P*
eGFR with cystatin-C-based CAPA (mL/min/1.73m^2^)	108 (93-123)								
Age (y)	13 (9-22)								
Age (y)			−244	−281 to −206	**<.001**		−181	−236 to −127	**<.001**
Age (y)^2^			53	15 to 90	**.006**		45	0.87 to 90	**0.046**
BMI (kg/m^2^)	20 (16-24)		−1.9	−2.3 to −1.4	**<.001**		−0.38	−0.95 to 0.20	.20
Ethnicity					.052				
Asian	20 (9.6)		—	—	−		—	—	
Black	15 (7.2)		17	−0.02 to 34	.050		23	10 to 35	**<.001**
White	168 (80)		15	2.9 to 27	**.015**		19	9.9 to 27	**<.001**
Other	6 (2.9)		26	3.1 to 50	**.027**		17	−0.89 to 34	.063
History of renal disease	16 (7.7)				**<.001**				
No			—	—			—	—	
Yes			−22	−35 to −9.1	**<.001**		−6.2	−16 to 3.8	.22
History of diabetes	2 (1.0)				**.004**				
No			−	−			—	—	
Yes			−52	−88 to −17	**.004**		−29	−55 to −2.9	**.029**
History of hypertension	21 (10)				**<.001**				
No			—	—			—	—	
Yes			−44	−54 to −34	**<.001**		−18	−30 to −6.8	**.002**
Blood pressure category (per AHA)					**<.001**				
Normal	122 (58)		−	−			—	—	
Elevated	35 (17)		−6.2	−15 to 2.1	.14		2.7	−4.8 to 10	.48
HTN-I	37 (18)		−25	−33 to −17	**<.001**		0.02	−8.7 to 8.7	>.99
HTN-II	15 (7.2)		−43	−55 to −31	**<.001**		−4.2	−16 to 8.0	.50
Severity group					.32				
Inhibitor	28 (13)		—	—			—	—	
No inhibitor: severe (<1%)	38 (18)		−5.0	−18 to 7.6	.44		−1.8	−11 to 7.5	.70
No inhibitor: moderate (1%-5%)	106 (51)		3.5	−7.3 to 14	.52		−2.8	−11 to 5.6	.52
No inhibitor: mild (>5%)	37 (18)		4.2	−8.5 to 17	.52		−7.0	−17 to 2.7	.16
NSAID	13 (6.2)				.18				
No			—	—			—	—	
Yes			−10	−24 to 4.5	.18		10	−1.0 to 21	.076

The forest plot displays the β coefficient and 95% Cs for each variable included in the univariate or multivariate linear regression models. Each point represents the β coefficient for a variable, with the horizontal lines indicating the 95% CI. The vertical dashed line indicates the null value (β = 0), where there is no effect. Bold values denote statistical significance at the *P* < .05 level.

AHA, American Heart Association; BMI, body mass index; eGFR, estimated glomerular filtration rate; HTN, hypertension; NSAID, nonsteroidal anti-inflammatory drug.

a Median (IQR) or *n* (%).

### 3.3 Barrier damage

The UACR values exhibited a high degree of right skewness (median, 0.13; IQR, 0.38; mean, 15.1; SD, 137.1; minimum, 0.00; and maximum, 1443.5). According to the KDIGO definition [11], 175 subjects (95% of the study cohort) had normal to mildly increased albuminuria (defined as UACR <3 mg/mmol); of these, 147 subjects had normal/high eGFR, 26 had mildly decreased eGFR and 2 had mildly to moderately decreased eGFR (Table 3). Seven patients with moderately increased albuminuria (UACR between 3 and 30 mg/mmol) had normal (*n* = 3) or mildly reduced (*n* = 4) eGFR (60-89 ml/min/1.73 m^2^). All 3 patients with severely increased albuminuria (UACR >30 mg/mmol) had mildly to moderately decreased eGFR (UACR, 34.9 mg/mmol; FIX inhibitor, and HTN-II); severely decreased eGFR (UACR, 1189 mg/mmol; FIX inhibitor, tubulointerstitial nephritis, and HTN-II); or kidney failure (UACR, 1443 mg/mmol; severe disease without FIX inhibitor, renal insufficiency, and HTN-I).

**Table 3:** Distribution of albuminuria categories by eGFR Categories.

eGFR CAPA category (KDIGO 2024)	Albuminuria category (KDIGO 2024)			
	Normal to mildly increased	Moderately increased	Severely increased	Total
Normal or high	147 (79)	3 (1.6)	0 (0)	150 (81)
Mildly to moderately decreased	2 (1.1)	0 (0)	1 (0.5)	3 (1.6)
Mildly decreased	26 (14)	4 (2.2)	0 (0)	30 (16)
Severely decreased	0 (0)	0 (0)	1 (0.5)	1 (0.5)
Kidney failure	0 (0)	0 (0)	1 (0.5)	1 (0.5)
Total	175 (95)	7 (3.8)	3 (1.6)	185 (100)

Values are *n* (%). Fisher exact test, *P* < .001.

eGFR, estimated glomerular filtration rate; KDIGO, Kidney Disease: Improving Global Outcomes.

The distribution of UIGCR was also skewed to the right (median, 0.26; IQR, 0.24; mean, 1.71; SD, 17.8; minimum, 0.07; and maximum, 242.9). Two subjects showed extreme UIGCR values (9.98 and 242.9 mg/mmol) and were the same 2 subjects who also had extreme UACR values (described earlier).

Results from the ranked regression analyses (Table 4) showed that none of the patient characteristics or clinical outcomes had significant associations with UACR. However, strong negative associations with UIGCR were indicated for age, BMI, Black vs Asian ethnicity, and elevated blood pressure compared with normal blood pressure (β, −0.41, −0.35, −0.46, and −0.25, respectively).

**Table 4:** Ranked regression analysis of the relationship between barrier damage and tubular dysfunction with patient demographics or clinical characteristics.

Characteristic	*n*	β	95% CI	*P*	β	95% CI	*P*	β	95% CI	*P*
Age (y)	185	−0.01	−0.15 to 0.14	.91	−0.41	−0.54 to −0.27	**<.001**	0.15	0.09 to 0.20	**<.001**
BMI (kg/m^2^)	185	0.00	−0.15 to 0.15	>.99						
Ethnicity	185									
Asian		—	—		—	—		—	—	
Black		6.9	−33 to 47	.74	−46	−85 to −7.0	**.021**	12	−4.5 to 28	.15
White		14	−17 to 45	.37	2.0	−28 to 32	.90	5.6	−7.0 to 18	.38
Other		−5.6	−58 to 47	.83	4.2	−47 to 55	.87	0.00	−21 to 21	>.99
History of renal disease	185									
No		—	—		—	—		—	—	
Yes		15	−13 to 44	.29	9.3	−19 to 38	.52	15	3.5 to 27	**.011**
History of diabetes	185									
No		—	—		—	—		—	—	
Yes		−20	−126 to 86	.71	54	−52 to 160	.31	−5.5	−49 to 38	.80
History of hypertension	185									
No		—	—		—	—		—	—	
Yes		24	−1.1 to 50	.061	19	−6.4 to 45	.14	38	29 to 47	**<.001**
Blood pressure category (per AHA)	185									
Normal		—	—		—	—		—	—	
Elevated		−15	−37 to 7.1	.18	−26	−49 to −4.2	**.020**	0.00	−8.2 to 8.2	>.99
HTN-I		19	−1.0 to 39	.063	−5.0	−25 to 15	.62	18	11 to 25	**<.001**
HTN-II		9.7	−23 to 43	.56	−11	−44 to 23	.53	34	21 to 46	**<.001**
Severity group	185									
Inhibitor		—	—		—	—		—	—	
No inhibitor: severe (<1%)		−19	−48 to 8.9	.18	−27	−55 to 1.4	.062	−9.0	−21 to 2.6	.13
No inhibitor: moderate (1%-5%)		−20	−43 to 4.2	.11	−17	−41 to 6.5	.15	−11	−21 to −1.4	**.025**
No inhibitor: mild (>5%)		−18	−46 to 10	.21	−21	−49 to 6.4	.13	−12	−23 to −0.85	**.035**
NSAID	185									
No		—	—		—	—		—	—	
Yes		−1.2	−33 to 30	.94	8.2	−23 to 40	.61	1.9	−11 to 15	.77
BMI (kg/m^2^)					−0.35	−0.49 to −0.21	**<.001**	0.10	0.04 to 0.16	**.001**

The beta regression coefficient is equivalent to the Spearman rank-order correlation coefficient, providing a measure of the monotonic relationship between ranked variables. Bold values denote statistical significance at the *P* < .05 level.

AHA, American Heart Association; BMI, body mass index; HTN, hypertension; NSAID, nonsteroidal anti-inflammatory drug; UACR, urine albumin/creatinine ratio; UIGCR, urine immunoglobulin/creatinine ratio.

### 3.4 Tubular dysfunction

Only 11 of the 185 patients (5.9%) with urinalysis data had measurable levels of protein HC, 5 of which were at the level of detection (2.5 mg/L). The values in the remaining 6 samples were between 7.5 and 285 mg/L (creatinine-adjusted values, 0.66-58.52), among which 4 were <12, indicating minor dysfunction. The 2 highest values in our cohort (182 and 285 mg/L, respectively; creatinine-adjusted values of 22.58 and 58.52, respectively) were found in patients with also severely increased UACR and significantly increased UIGCR, as described earlier. Ranked regression (Table 4) showed strong associations between protein HC and age (β, 0.15), BMI (β, 0.10), a history of kidney disease (β, 15), HTN-I and HTN-II vs normal blood pressure (β, 18 and 34, respectively), and mild and moderate disease vs FIX inhibitor (β, −11 and −12, respectively).

Ranked regression analysis was conducted to evaluate the relationship between the damage markers or tubular dysfunction, and eGFR. The β coefficient for UACR was negative (β, −0.04; 95% CI, −0.19 to 0.10) and was not statistically significant (*P* = .57). UIGCR showed a positive and statistically significant association with eGFR. The β coefficient was positive (β, 0.19; 95% CI, 0.05-0.33), and the result was significant (*P* = 0.010). This indicates that as UIGCR increases, there is a corresponding increase in eGFR. The β coefficient for protein HC was negative (β, −0.76; 95% CI, −1.1 to −0.42) and statistically significant (*P* < .001). This indicates a significant negative association between protein HC and eGFR, suggesting that higher values of protein HC are associated with lower values of eGFR.

## 4 Discussion

This observational study focusing on patients with HB showed no evidence that reduced glomerular filtration rate, glomerular protein leakage, and tubular dysfunction are more common in patients with HB without inhibitors compared with those in the general population, despite their increased bleeding tendency and/or the hemophilia treatments they receive. As expected, and seen also in the general population, factors such as diabetes, kidney diseases, advanced age, and high blood pressure were significantly correlated with reduced filtration rate, suggesting that the same risk factors for kidney impairment apply to patients with HB as well. Subgroup analyses further demonstrated no differences in kidney function between patients with severe and nonsevere forms of HB. Concerns have been raised among physicians treating persons with severe hemophilia about the potential for regular replacement therapy to trigger an immune response due to the patients’ long-term exposure to its protein loads, and this in turn could lead the formation of immune complexes and possibly causing damage to the glomerular filtration barrier over time. However, we found no evidence of this in our study, as patients receiving regular prophylaxis had comparable filtration rates to those treated on-demand. Furthermore, patients with inhibitors—all with a severe form of the disease—also demonstrated similar kidney function, even if they had undergone one or more courses of ITI therapy, unless they had a reported pre-existing kidney disease. Importantly, however, the subgroup of patients with inhibitors having performed ITI is relatively small, and based on the retrospective study design, no unequivocally interpretation can be made.

Another potential risk in persons with an increased bleeding tendency is whether bleeds in the kidney tissue including clinical or subclinical hematuria, could negatively affect glomerular and/or tubular function. However, consistent with previous studies, our data do not support this notion. Patients in our cohort receiving on-demand treatment exhibited similar eGFR and tubular function compared with those on regular prophylaxis, even when having severe HB. One interesting observation, however, was the relatively lower filtration rate seen in patients of Asian ethnicity, despite their generally younger age. It is well-known that individuals of Black ethnicity are genetically predisposed to a higher risk of renal failure [17]. There are also data indicating that Asian Americans have more frequent early renal damage based on UACR than the White US population [18]. However, no current data suggest that Asian populations are at increased risk for reduced glomerular filtration rate than other ethnicities [18]. In our study, the Asian patients were infrequently treated with prophylaxis, likely due to the high cost of treatment, which theoretically put them at a higher risk of bleeds in any tissue. This raises the possibility that bleeding episodes may still have an effect on glomerular filtration rate (GFR). Unfortunately, we lacked precise bleed data for all patients in the cohort, preventing a more accurate evaluation of this potential impact, which warrants further investigation. We used the CAPA formula [9], as this is developed among both children and adults. Furthermore, it is recognized as a validated eGFR formula in the KDIGO guidelines [11]. Additionally, while the CAPA formula [9], used to estimate GFR, was developed using White and Japanese populations, it has not been validated across all ethnic groups. Therefore, the findings regarding the Asian subgroup in our study should be interpreted with caution, but further evaluated.

Our study has both limitations and strengths. One notable limitation is the absence of a control group. However, no abnormal findings were observed concerning eGFR and UACR when using standard methods and algorithms, which reduces the necessity for a control group [19]. Moreover, although our cohort represents one of the larger studies in the field of hemophilia, particularly HB, the sample size remains relatively small, which could influence the robustness of the findings and their interpretations, not least in the oldest subgroups, since the majority of patients (64%) were younger than 18 years of age. Sampling for the evaluation of renal function and injury was only performed at study entry and correlated to the medical history and physical parameters including the blood pressure measured at the visits. Therefore, fluctuations over time were not considered, and a more longitudinal approach would have been valuable. Because we basically relied on historical data, this retrospective approach carries the risk of missing or biased information, which could impact the study’s outcome. The close follow-up of PWH and a more holistic approach to the care in many centers over the years would, however, probably partly compensate for these weaknesses. Measured GFR would be desired but hard to achieve in large cohorts and especially among children.

A key strength of our study was the prospective 6-month follow-up period after enrollment along with the large cohort of patients with HB. Most studies in the hemophilia field, including those assessing kidney function, have focused on HA, with research on HB being both rare and typically involving very small sample sizes. Given the limited number of eligible patients at each center, nearly all patients who met the inclusion criteria were enrolled, minimizing recruitment bias. Another strength is the central testing of all samples and the combined use of both plasma and urine samples, which is uncommon in many studies. UACR is a particularly strong marker for kidney disease and glomerular damage, often appearing before a decline in GFR. Additionally, for the calculations of eGFR, we used cystatin C instead of the more commonly used creatinine, recognizing its advantages in pediatric populations. Furthermore, the eGFR-formula CAPA, used in this study, was constructed from both Whites and Asians. This allowed us to compare filtration rates across ages in both children and adults. Since many PWH experience arthropathies and secondary muscular atrophy, the use of creatinine-based formulas would have been less accurate, further justifying our choice of cystatin C.

In conclusion, our study found no evidence that White patients with HB and no inhibitors, regardless of disease severity or whether they receive regular treatment with factor concentrates, are at an increased risk of developing low GFR, glomerular damage, or tubular dysfunction compared with the general population. Age and hypertension emerged as the most significant risk factors, underscoring the importance of monitoring these common risk factors closely in PWH, just as in individuals without hemophilia.
